# The Effect of Small Sample Size on Measurement Equivalence of Psychometric Questionnaires in MIMIC Model: A Simulation Study

**DOI:** 10.1155/2017/7596101

**Published:** 2017-06-20

**Authors:** Jamshid Jamali, Seyyed Mohammad Taghi Ayatollahi, Peyman Jafari

**Affiliations:** Department of Biostatistics, Faculty of Medicine, Shiraz University of Medical Sciences, Shiraz, Iran

## Abstract

Evaluating measurement equivalence (also known as differential item functioning (DIF)) is an important part of the process of validating psychometric questionnaires. This study aimed at evaluating the multiple indicators multiple causes (MIMIC) model for DIF detection when latent construct distribution is nonnormal and the focal group sample size is small. In this simulation-based study, Type I error rates and power of MIMIC model for detecting uniform-DIF were investigated under different combinations of reference to focal group sample size ratio, magnitude of the uniform-DIF effect, scale length, the number of response categories, and latent trait distribution. Moderate and high skewness in the latent trait distribution led to a decrease of 0.33% and 0.47% power of MIMIC model for detecting uniform-DIF, respectively. The findings indicated that, by increasing the scale length, the number of response categories and magnitude DIF improved the power of MIMIC model, by 3.47%, 4.83%, and 20.35%, respectively; it also decreased Type I error of MIMIC approach by 2.81%, 5.66%, and 0.04%, respectively. This study revealed that power of MIMIC model was at an acceptable level when latent trait distributions were skewed. However, empirical Type I error rate was slightly greater than nominal significance level. Consequently, the MIMIC was recommended for detection of uniform-DIF when latent construct distribution is nonnormal and the focal group sample size is small.

## 1. Introduction

In recent years, the use of differential item functioning (DIF) has also been referred to as measurement equivalence, which has been widely used to validate psychological assessment instruments such as quality of life [1, 2]. People with the same quality of life level should be able to answer the items in the quality of life questionnaire the same way, regardless of their education, gender, or other group memberships [3]. Mean score in the quality of life may differ among groups and DIF occurs when an item in the questionnaire has different measurement properties for one group of individuals versus another, irrespective of the mean differences on the construct [4].

Several methods have been developed for identifying DIF in test items. All DIF detection methods fall under the parametric and nonparametric methods. Mantel-Haenszel, standardization, and simultaneous item bias test are important nonparametric methods while item response theory, logistic and ordinal logistic regression, multiple-group analysis, and multiple indicators multiple causes (MIMIC) are important parametric methods for DIF testing [5].

Multiple-group analysis and MIMIC are two approaches of structural equation modeling, which have been widely used to assess DIF by many applied researches [6–8]. Previous studies have shown that, under specific conditions, multiple-group analysis is preferable to the MIMIC approach [4], but multiple-group analysis requires a large sample size. This is due to the fact that the model will fit to data for each group separately [4]. In this study, we have merely focused on MIMIC model as a well-known method to detect DIF [4]. The MIMIC model has several advantages when compared with other methods of DIF detection; it requires smaller sample size, latent variables can be predicted by at least one single-item indicator, it can be applied for dichotomous and polytomous items, it is not necessarily used for all items with the same number of response categories, and it provides information on the structural and measurement models [1, 4, 9, 10].

Previous simulation studies have investigated some MIMIC model properties in DIF detection including the structure of data, the scale of response categories, DIF pattern, differences in mean, and variance of latent trait distribution [4, 9–17].

Typically, in DIF testing of medical studies, two groups are assumed to be labeled as the reference and focal groups, where patients are often placed in the latter group. A common problem in medical and psychological studies is the small sample size, particularly in the focal group, where access to patients or rare disease patients is difficult. Furthermore, the small sample size prevents wasting of time and money [18]. Consequently, evaluation of statistical properties of MIMIC model for detecting DIF can be quite valuable when the focal group is small.

Skewness of latent trait distribution, also referred to as latent construct, is an important point that needs to be considered in DIF detection [19, 20]. In psychological investigations, it is possible to be confronted with nonnormal distribution cases. Several researchers have discussed the statistical properties of MIMIC model in DIF testing with actual data [21]. They concluded that the use of different methods for evaluating DIF may lead to different results. To the best of our knowledge, the skewness of latent trait distributions in DIF detection by MIMIC model has not been investigated.

A Monte Carlo simulation study is an essential tool for assessing the behavior of MIMIC model under various conditions. This study is the first simulation-based investigation to assess MIMIC model for DIF detection, when latent construct distribution is nonnormal and focal sample size is small. We have discussed the advantages and disadvantages through a series of simulations.

## 2. Method

### 2.1. MIMIC DIF Detection

Two types of DIF can be identified and denoted as uniform and nonuniform [22]. Uniform-DIF is the simplest type of DIF where the probability of selecting the specific category of item is greater (or lesser) for one group than the other in all levels of the latent construct uniformly. Uniform-DIF occurs when item difficulty parameters are different in the two groups [22]. Nonuniform-DIF transpires when the probability of answering a specific category of items among groups varies at all latent construct levels [22].

**Figure 1:**
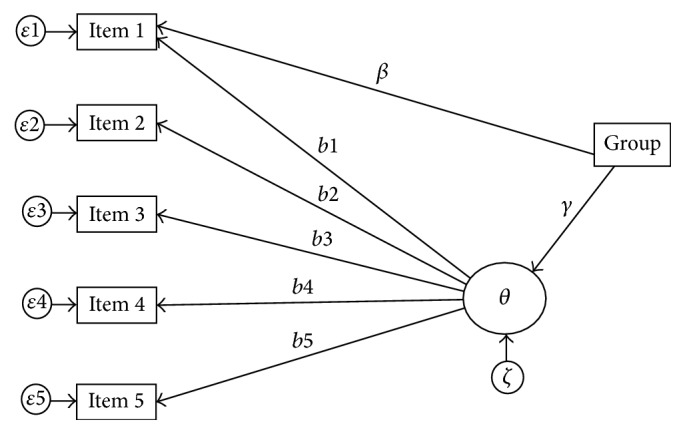
A MIMIC model for detecting uniform-DIF in 5-item scale. Rectangles are observed variables; circles are latent variables; *γ*: the regression coefficient displaying the mean difference on the latent trait; *b*_*i*_: threshold parameter; *β*: the regression coefficient displaying the group difference in the threshold for item *i* and the grouping variables; *ɛ*_*i*_: the measurement error for item *i*; *ζ*: a residual for latent trait (*θ*).* Note*. Item 2 to item 5 constitute the DIF free, when item 1 is tested for uniform-DIF.

Uniform-DIF detection with MIMIC model is performed with regressing potential DIF items and latent variable (*θ*) onto a covariate concurrently [14]. This covariate can be either continuous or categorical in nature, but usually in medicine and psychological research, it is assumed as dichotomous variable. The mechanism of MIMIC for detection of uniform-DIF is shown in Figure 1. Nonuniform-DIF can be assessed by regressing the interaction between the latent factor (*θ*) and the group membership indicator (Xi) on potential DIF items [15]. Although the MIMIC model is a useful approach for identifying uniform-DIF, the accuracy of this model in detecting nonuniform-DIF appeared to be questionable [23]. In this study, we have only focused on uniform-DIF, which is important among applied researchers.

### 2.2. Data Generation

Ordinal responses were generated from the graded response model (GRM) [24]:

(1) $$\begin{array}{c} p_{ij}\left(\;\theta \;\right)=\frac{e^{a_{i}\left(\theta -b_{ij}\right)}}{1+e^{a_{i}\left(\theta -b_{ij}\right)}}, \end{array}$$

where *p*_*ij*_(*θ*) is the probability of a respondent selecting a particular response category (*j*) or above category for one item (*i*), *a*_*i*_ and *b*_*ij*_ are the discrimination and threshold parameters, and *θ* denotes the latent trait. Determining the distribution of discrimination parameters was carried out based on empirical research and preliminary simulation. In all conditions, *a*_*i*_ and *b*_*ij*_ were drawn from the uniform distribution over the 1.5 and 2 interval and standard normal distribution, respectively.

### 2.3. Simulation Scenarios

In this study, we have assumed two groups that were labeled as reference and focal groups. The five factors in this simulation study were investigated: reference to focal group sample size ratio, magnitude of the uniform-DIF effect, scale length, the number of response categories, and latent trait distribution.

Sample size ratio between the reference and focal groups was set at R100/F100, R200/F100, R300/F100, R400/F100, and R500/F100. Medium and severe uniform-DIF were also simulated by adding 0.5 and 1 to *b*_*ij*_ parameters to the focal group, respectively [3, 25]. The length of the scale was considered 5 and 10. It is worth mentioning that Likert-type scales and odd number response categories are frequently used in psychological and medical research. In this simulation study, 3-, 5-, and 7-point ordinal responses were used and all items had the same number of response categories. To evaluate the impact of the latent construct distribution on DIF detection with MIMIC model, we simulated six different distribution conditions (Table 1). In previous studies, MIMIC model properties in DIF detection were investigated when the latent trait distribution was assumed normal [4, 26, 27]. In this study, we used Beta distribution to generate skewed latent construct distributions. Beta (1, 4) and Beta (0.5, 4) distributions were used for situations in which the participants responded moderately and mostly negatively, and Beta (4, 1) and Beta (4, 0.5) were used when they responded moderately and highly positively [3]. Since the generated data by Beta distribution with considered parameters are into the (0,1), then to compare with the standard normal distribution, we should standardize it.

In total, we generated 780 (5*∗*2*∗*2*∗*3*∗*13) independent simulation situations; each simulated condition was simulated 1000 times.

Nonconvergence situation is one of the most common problems during estimation in MIMIC model. The small sample size, not positive definite matrices, and out of bounds estimates are three important causes of nonconvergence situation in MIMIC model [28, 29]. Out of bounds estimates are sometimes referred to as “Heywood cases” when either improper solutions for standard error/variance (less than 0) or improper solutions for correlation (greater than 1 or less than −1) occur [28]. In this study, the number of convergence replications was calculated. In the present study, seed was used to control the randomness error of the random number generation [30]. Harwell et al. emphasized the use of seed in the simulation study that can lead to minimizing the effect of random error on parameter estimates [31]. Another advantage by determining seed is that it will be easy to reproduce the same data set afterward, which might require to be reviewed later [31]. To achieve reliable results, if the number of convergences was low, the seed given to the R program was changed and the analysis was repeated.

Statistical power is defined by the ratio of the number of times DIF was correctly identified by MIMIC method across replications. For calculating the power, we have assumed that item 1 has uniform-DIF. The Type I error rate, also referred to as false positives, was assessed by the proportion of times that DIF was incorrectly identified in the 1000 replications [32].

The CatIrt and Lavaan packages in R version 3.21 software were used to generate data from GRM model and fitting MIMIC model for DIF testing, respectively [33, 34]. The nominal Type I error rate for this study was 0.05.

## 3. Results

### 3.1. Effect of Reference to Focal Group Sample Size Ratio on Detecting Uniform-DIF

By increasing the sample size, the power of MIMIC model was systematically increased; however, there was no pattern in Type I error. The results of this study showed that when latent trait distribution in the reference group was the standard normal or latent trait distribution in the reference and focal groups was the same, a sample size of 500 for graded items with 3 ordered categories of response (R400/F100) and 300 for items with 5 and 7 categories of response (R200/F100) suffices. Refer to Tables 3 and 5 for more information on mutations in power and Type I error rate.

### 3.2. Effect of Magnitude of DIF on Detecting Uniform-DIF

When other circumstances stayed fixed, increase in the magnitude of DIF led to improved MIMIC model power in detecting uniform-DIF: 20.35% in total and 24.28% and 16.42% in increasing the magnitude of DIF from medium to severe in 5-item and 10-item scales, respectively. In such situation, Type I error did not change significantly.

### 3.3. Effect of Scale Length on Detecting Uniform-DIF

Increasing the scale length from 5 to 10 items caused an increase of approximately 3.47% in the power of MIMIC model for detecting uniform-DIF. According to our results, increase in the number of items from 5 to 10 led to improvement of the MIMIC model power for detecting medium uniform-DIF: 6.79% in total, 8.78% in 3-point response scale, 5.90% in 5-point response scale, and 5.71% in 7-point response scale. In this situation, Type I error rate was changed slightly (2.76%).

Increase in the number of items from 5 to 10 led to decreased Type I error rate of MIMIC model for detecting severe uniform-DIF: 2.87% in total, 1.56% in 3-point response scale, 1.01% in 5-point response scale, and 6.03% in 7-point response scale. In this circumstance, the power was changed about 0.15%.

### 3.4. Effect of Number of Response Categories on Detecting Uniform-DIF

When other conditions remained constant, increase in the number of response categories led to improved MIMIC model power in detecting uniform-DIF: 4.83% in total and 5.66%, 1.52%, and 7.33% in increasing the number of response categories from 3 to 5, from 5 to 7, and from 3 to 7, respectively.

Simultaneously, when other conditions were fixed, increasing the number of response categories led to a decrease in the Type I error MIMIC model in detecting uniform-DIF: 5.66% in total and 2.73%, 5.47%, and 8.80% in increasing the number of response categories from 3 to 5, from 5 to 7, and from 3 to 7, respectively.

### 3.5. Effect of Latent Trait Distribution on Detecting Uniform-DIF

Skewness in the latent trait distribution led to a slight change in the magnitude of Type I and power of MIMIC model for detecting uniform-DIF. When latent trait distributions were normal (condition 13), moderate (conditions 1, 3, 5, 7, 9, and 11), and highly skewed (conditions 2, 4, 6, 8, 10, and 12), mean powers of MIMIC model to detect uniform-DIF were 0.920, 0.917, and 0.915; with Type I error, they were 0.054, 0.059, and 0.069, respectively. When latent trait distributions were normal, moderate, and highly skewed, mean powers of MIMIC model to detect medium uniform-DIF were 0.842, 0.837, and 0.835; with Type I error, they were 0.054, 0.059, and 0.069, respectively. When latent trait distributions were normal, moderate, and highly skewed, mean powers of MIMIC model to detect severe uniform-DIF were 0.998, 0.997, and 0.995; with Type I error, they were 0.054, 0.060, and 0.069, respectively.

In most scenarios, when latent trait in the reference group was normal distribution or latent trait distribution in the reference and focal groups was the same (all conditions except 10 and 12), Type I error was less than 0.06 and power of MIMIC model was at an acceptable level (greater than 80%). Therefore, we can conclude that MIMIC model had a robust to skewness in latent trait. In conditions 10 and 12, when latent trait distribution in one group was highly positively skewed and in another group was highly negatively skewed or vice versa. MIMIC model was at its lowest power and the greatest Type I error in discovering uniform-DIF.

We performed all 390 different scenarios' simulation for the small magnitude of DIF (magnitude of DIF was 0.25). Under the best circumstances, when we had larger sample size (R500/F100), the 10-item scale, severe uniform-DIF, 7-point ordinal responses, and the latent trait distribution in both groups were normal, and power and Type I error were 0.489 and 0.055, respectively. So given that the MIMIC model was not appropriate for detecting small uniform-DIF, we refrained from describing the results.

All 1000 replications met the convergence criteria when latent trait distribution had a normal or skewed distribution. In all scenarios, goodness-of-fit indices such as Root Mean Square Error of Approximation (RMSEA), Root Mean squared Residual (RMR), Tucker–Lewis Index (TLI), Comparative Fit Index (CFI), and Goodness-of-Fit Index (GFI) were in an acceptable level. Space management prevented us from presenting the results for goodness of fit for all the simulation in detail.

Tables 2 and 3 show the Type I error rates and power of MIMIC model for detecting uniform-DIF in 5-item scale. Tables 4 and 5 indicate the statistical properties of MIMIC model for detecting uniform-DIF in 10-item scale.

### 3.6. Real Data Example

In this section, we explain the example of the questionnaire to assess the effect of small sample size on measurement equivalence of psychometric questionnaires in the MIMIC model.

The 12-item General Health Questionnaire (GHQ-12) is an appropriate instrument to assess Minor Psychiatric Disorders (MPD) during the previous month [35]. A cross-sectional study was conducted to identify the MPD with GHQ-12 among 771 nurses employed in hospitals of the Fars and Bushehr provinces, Southern Iran, between October and December 2014. Only a brief description of the data used in this study is mentioned here because they have been fully described elsewhere [35].

Of the 269 men participating in the study, 100 men were randomly selected. Among 502 women, samples with the size of 100, 200, 300, 400, and 500 were randomly chosen.

The results of fitting the MIMIC model to detect uniform-DIF are shown in Table 6. In all the sample sizes, item 12 of the GHQ-12 was detected with uniform-DIF. The intensity of uniform-DIF for item 12 was severe and for item 1 was medium. For this reason, in large sample size (M100/F400 and M100/F500) item 1 of the GHQ-12 was detected with uniform-DIF with the MIMIC model.

## 4. Discussion

The present study provided a simulation-based framework to determine the statistical properties of MIMIC model when latent trait distribution was nonnormal and sample size was small.

Up to now, in most simulation researches, item responses were produced using the GRM when latent trait was normally distributed. However, in many psychological researches, the assumption of normality latent construct can frequently be violated in practice [36, 37]. What distinguishes this study from previous ones was the effort to assess the performance of MIMIC model in uniform-DIF detection when latent trait distribution was nonnormal. Our results showed that skewness in the latent trait distribution cannot affect MIMIC model performance in uniform-DIF detection. However, Type I error inflated when latent trait distribution in one group was highly positively skewed and in another group it was highly negatively skewed or contrariwise. Until now, there has been no documented evidence that has investigated the effect of skewness of latent construct distribution on the performance of MIMIC model. However, Monaco found that high skewness in latent trait distribution resulted in a 5% to 10% decrease in the power for detecting DIF in dichotomous items in the differential functioning of items and tests, Mantel-Haenszel, and Lord's chi-square methods [38]. The research carried out by Kaya et al. concluded that moderate skewness in latent trait leads to approximately 10% decrease in the power for detecting uniform-DIF by logistic regression in polytomous items [20]. Another Monte Carlo simulation study showed that high skewness in latent trait distribution could reduce the power ordinal logistic regression model up to 57.7% [3].

Under various combinations of latent trait distributions, the power of MIMIC model increased as the reference group sample size increased, but Type I error did not obey a specific pattern. This finding is consistent with those of previous studies that demonstrated when sample size increased, the power for detecting DIF increased [4, 23]. The unbalanced sample sizes between the focal and the reference group are popular in real-life circumstances. In previous simulation studies, sample size ratio between the focal and reference groups varied between 1 and 5 [4, 9, 27]. A previous study indicated that MIMIC model DIF detection test was not powerful enough when the sample size ratio between the focal and reference groups was smaller than 5 (R500/F100), and latent trait was the normal distribution [4]. However, we found that, in these situations, the MIMIC model was powerful for detecting uniform-DIF when sample size ratio was more than 3 (R300/F100) in 3-point response scale and more than 2 (R200/F100) in 5- or 7-point response scale.

The results from a research study indicated that increasing the number of items could lead to improvement in the power and decrease in the Type I error rate of MIMIC model for detecting uniform-DIF. With respect to this, our results were in line with the results of several studies [4, 9, 25, 39]. However, few researchers have argued that the number of indicators does not appear to affect the power [14].

When the magnitude of uniform-DIF was increased, the performance of MIMIC model improved; that is, the power increased and Type I error was reduced. This was an expected result, and similar results were reported in other studies [14, 40].

Another important feature considered in this study was evaluation of the number of response categories that could affect the power of MIMIC model for detecting DIF. Our study shows that increased number of response categories resulted in a systematic increase in the power of MIMIC model for detecting uniform-DIF. By increasing the number of items from 5 to 7, the MIMIC model power improved just 1.52% for detection of uniform-DIF. Increasing the number of response categories creates problems for low educated participants; hence, we suggested 5-point response scale that was more suitable for people with lower levels of education which was easier to interpret. Allahyari et al. recommended the minimum number of response categories for DIF analysis to be five [3]. Willse and Goodman in a simulation study showed that MIMIC model for continuous variables had better performance than categorical variables for DIF testing [39].

Our study showed that the number of convergence MIMIC models did not depend on the skewness rate in latent construct distribution. In numerical analysis, the number of convergences could be affected by the method used for parameter estimation [39, 41]. There are several methods for parameter estimation in MIMIC model, including maximum likelihood (ML), generalized least squares (GLS), weighted least squares (WLS), weighted least squares means, and variance adjusted (WLSMV). In this study, ML was used for parameter estimation. Previous studies have demonstrated that ML method was preferable to the GLS and WLS procedures when data were nonnormal in MIMIC model [42]. Another previous study showed that the ML method has less Type I error than the WLSMV [43]. Also, GLS and WLS require a larger sample size than ML estimation for the fitting model [39].

MIMIC model uses single latent covariance matrix for parameter estimation. Hence, in this model, it is assumed that the variance of latent factor is equal across the groups. Carroll concluded that violating the homogeneity of variance assumption could lead to inflated Type 1 error in DIF detection and increase in bias in estimating the factor loadings and the latent group mean difference [14]. Our study showed that the heterogeneity of variance (conditions 1, 2, 3, 4, 9, 10, 11, and 12) led to an increase in Type I error MIMIC model in detection of uniform-DIF.

There are many different methods to make DIF items. The most common technique for generating DIF items is adding a certain amount to all thresholds for the focal group which was used in this study. Although this issue is controversial, some authors point out that, by adding or subtracting a value asymmetrically to the parameters threshold, this action could affect performance model for DIF detection [3]. Scott et al. indicated that reducing or adding a specified amount to the threshold does not affect the results significantly [25].

Finally, this study had some limitations which need to be taken into account. Previous simulation studies have shown that power of MIMIC model could be affected by the number of DIF items [11]. On the contrary, in this study, we have assumed that there is only one item which has uniform-DIF. If this condition was taken into consideration, we were forced to simulate a larger number of scenarios, which was time-consuming. The MIMIC model can be used for both uniform and nonuniform-DIF detection. However, most researchers believe that MIMIC model is not an appropriate performance to detect nonuniform-DIF, because the parameterization of the MIMIC model was only suitable for identifying uniform-DIF [23, 44]. Also, nonuniform-DIF has computational effort required to fit MIMIC model because the latent trait cannot be simply multiplied by the group variable which is an observed variable [15]. In this study, we limited our DIF detection to uniform-DIF and two groups at a time, a reference group and a focal group. Nonetheless, the MIMIC model can handle two types of DIF and more than two groups [15, 21].

## 5. Conclusion

Our findings showed that, by increasing the number of response categories, the number of items, the magnitude of DIF, and sample size could lead to an increase in power of MIMIC model for uniform-DIF detecting. This study revealed that MIMIC model in detection of uniform-DIF was fairly robust to departure from the normal latent trait distribution assumption. When latent trait distributions were skewed, the power of MIMIC model in detection of uniform-DIF was at an acceptable level. However, empirical Type I error rate was slightly greater than nominal significance level of 0.05. Consequently, this technique is appropriated for uniform-DIF detection when latent trait distribution is nonnormal and the focal group sample size is small. Due to the insignificant effect on improving power by increasing the number of response categories from 5 to 7, we recommend 5-point response scale for uniform-DIF detection using MIMIC model, especially for participants with low levels of education. The results obtained from this study provide an appropriate guideline for further research. We recommend further studies to investigate the effect of the number of items with DIF and type of DIF on MIMIC model power when latent trait is skewed.

## Figures and Tables

**Table 1:** Distributions intended for latent trait in the reference and focal groups.

Condition	Ability distribution	
	Reference group	Focal group
1	*N* (0, 1)	Beta (1, 4)
2	*N* (0, 1)	Beta (0.5, 4)
3	*N* (0, 1)	Beta (4, 1)
4	*N* (0, 1)	Beta (4, 0.5)
5	Beta (1, 4)	Beta (1, 4)
6	Beta (0.5, 4)	Beta (0.5, 4)
7	Beta (4, 1)	Beta (4, 1)
8	Beta (4, 0.5)	Beta (4, 0.5)
9	Beta (1, 4)	Beta (4, 1)
10	Beta (0.5, 4)	Beta (4, 0.5)
11	Beta (4, 1)	Beta (1, 4)
12	Beta (4, 0.5)	Beta (0.5, 4)
13	*N* (0, 1)	*N* (0, 1)

**Table 2:** The mean of Type I error rates and power of MIMIC model for detecting medium uniform-DIF in 5-item scale.

	Distributions for *θ*	3 categories		5 categories		7 categories		Distributions for *θ*	3 categories		5 categories		7 categories	
		Power	Alpha	Power	Alpha	Power	Alpha		Power	Alpha	Power	Alpha	Power	Alpha
R100/F100	R: N (0, 1) F: Beta (1, 4)	0.637	0.086	0.688	0.065	0.755	0.057	R: N (0, 1) F: Beta (0.5, 4)	0.615	0.061	0.711	0.057	0.719	0.064
R200/F100		0.701	0.051	0.800	0.061	0.837	0.041		0.699	0.085	0.812	0.076	0.853	0.064
R300/F100		0.750	0.057	0.857	0.052	0.861	0.055		0.747	0.080	0.837	0.064	0.876	0.070
R400/F100		0.794	0.062	0.866	0.047	0.886	0.046		0.786	0.069	0.855	0.056	0.890	0.060
R500/F100		0.784	0.065	0.889	0.056	0.917	0.057		0.789	0.075	0.886	0.069	0.901	0.041

R100/F100	R: N (0, 1) F: Beta (4, 1)	0.638	0.058	0.698	0.059	0.759	0.060	R: N (0, 1) F: Beta (4, 0.5)	0.658	0.063	0.744	0.061	0.790	0.058
R200/F100		0.748	0.059	0.845	0.064	0.874	0.055		0.779	0.075	0.833	0.074	0.873	0.072
R300/F100		0.809	0.063	0.884	0.060	0.887	0.064		0.801	0.057	0.874	0.060	0.905	0.058
R400/F100		0.831	0.044	0.871	0.047	0.916	0.058		0.808	0.069	0.894	0.059	0.911	0.072
R500/F100		0.819	0.053	0.910	0.060	0.922	0.040		0.846	0.064	0.910	0.059	0.926	0.056

R100/F100	R: Beta (1, 4) F: Beta (1, 4)	0.616	0.053	0.725	0.050	0.710	0.057	R: Beta (0.5, 4) F: Beta (0.5, 4)	0.612	0.045	0.690	0.063	0.723	0.067
R200/F100		0.729	0.047	0.820	0.056	0.866	0.069		0.738	0.050	0.825	0.056	0.832	0.054
R300/F100		0.752	0.046	0.858	0.046	0.860	0.048		0.757	0.046	0.852	0.057	0.876	0.046
R400/F100		0.775	0.057	0.875	0.050	0.877	0.051		0.775	0.051	0.862	0.053	0.882	0.039
R500/F100		0.815	0.050	0.883	0.046	0.899	0.035		0.785	0.044	0.878	0.050	0.895	0.042

R100/F100	R: Beta (4, 1) F: Beta (4, 1)	0.639	0.049	0.731	0.061	0.750	0.057	R: Beta (4, 0.5) F: Beta (4, 0.5)	0.663	0.044	0.743	0.044	0.772	0.062
R200/F100		0.761	0.053	0.842	0.057	0.870	0.066		0.754	0.054	0.865	0.063	0.848	0.041
R300/F100		0.775	0.053	0.893	0.050	0.886	0.046		0.811	0.057	0.902	0.058	0.905	0.057
R400/F100		0.802	0.048	0.888	0.055	0.907	0.050		0.805	0.057	0.907	0.046	0.923	0.039
R500/F100		0.833	0.051	0.895	0.059	0.938	0.045		0.845	0.046	0.908	0.053	0.933	0.047

R100/F100	R:Beta (1, 4) F: Beta (4, 1)	0.608	0.073	0.723	0.054	0.739	0.069	R: Beta (0.5, 4) F: Beta (4, 0.5)	0.650	0.089	0.709	0.070	0.735	0.080
R200/F100		0.748	0.069	0.835	0.060	0.869	0.070		0.738	0.115	0.848	0.079	0.836	0.062
R300/F100		0.767	0.071	0.856	0.072	0.871	0.058		0.779	0.120	0.868	0.084	0.900	0.074
R400/F100		0.778	0.083	0.875	0.079	0.897	0.075		0.784	0.114	0.866	0.084	0.902	0.058
R500/F100		0.836	0.077	0.877	0.074	0.920	0.055		0.809	0.101	0.878	0.092	0.909	0.084

R100/F100	R: Beta (4, 1) F: Beta (1, 4)	0.582	0.054	0.685	0.073	0.711	0.071	R: Beta (4, 0.5) F: Beta (0.5, 4)	0.587	0.078	0.654	0.080	0.699	0.080
R200/F100		0.708	0.085	0.803	0.074	0.839	0.074		0.685	0.100	0.769	0.084	0.803	0.062
R300/F100		0.724	0.075	0.845	0.062	0.868	0.055		0.716	0.103	0.819	0.106	0.853	0.070
R400/F100		0.754	0.091	0.858	0.062	0.881	0.050		0.728	0.119	0.854	0.082	0.857	0.070
R500/F100		0.763	0.087	0.872	0.073	0.902	0.050		0.744	0.131	0.849	0.096	0.885	0.069

R100/F100	R: N (0, 1) F: N (0, 1)	0.609	0.058	0.708	0.055	0.770	0.066							
R200/F100		0.705	0.052	0.828	0.049	0.855	0.053							
R300/F100		0.785	0.060	0.852	0.057	0.896	0.048							
R400/F100		0.830	0.045	0.878	0.067	0.895	0.043							
R500/F100		0.803	0.060	0.881	0.062	0.900	0.046							

**Table 3:** The mean of Type I error rates and power of MIMIC model for detecting severe uniform-DIF in 5-item scale.

	Distributions for *θ*	3 categories		5 categories		7 categories		Distributions for *θ*	3 categories		5 categories		7 categories	
		Power	Alpha	Power	Alpha	Power	Alpha		Power	Alpha	Power	Alpha	Power	Alpha
R100/F100	R: N (0, 1) F: Beta (1, 4)	0.968	0.084	0.994	0.064	0.998	0.057	R: N (0, 1) F: Beta (0.5, 4)	0.963	0.060	0.992	0.055	0.991	0.063
R200/F100		0.982	0.055	0.998	0.061	0.999	0.042		0.980	0.084	1.000	0.076	1.000	0.065
R300/F100		0.991	0.056	1.000	0.052	1.000	0.055		0.986	0.078	1.000	0.065	1.000	0.070
R400/F100		0.990	0.062	0.998	0.046	1.000	0.046		0.979	0.069	0.999	0.054	1.000	0.059
R500/F100		0.989	0.066	1.000	0.056	1.000	0.059		0.987	0.074	0.999	0.070	1.000	0.041

R100/F100	R: N (0, 1) F: Beta (4, 1)	0.990	0.059	0.997	0.058	1.000	0.061	R: N (0, 1) F: Beta (4, 0.5)	1.000	0.065	0.997	0.061	0.998	0.058
R200/F100		0.999	0.059	1.000	0.062	1.000	0.057		0.999	0.073	1.000	0.074	1.000	0.071
R300/F100		0.998	0.063	1.000	0.059	1.000	0.063		0.999	0.057	1.000	0.060	1.000	0.058
R400/F100		0.999	0.045	1.000	0.047	1.000	0.059		0.999	0.069	1.000	0.060	1.000	0.072
R500/F100		0.998	0.052	1.000	0.060	1.000	0.040		1.000	0.064	1.000	0.060	1.000	0.056

R100/F100	R: Beta (1, 4) F: Beta (1, 4)	0.972	0.049	0.997	0.048	0.998	0.056	R: Beta (0.5, 4) F: Beta (0.5, 4)	0.966	0.042	0.989	0.063	0.997	0.067
R200/F100		0.990	0.047	0.999	0.057	0.999	0.067		0.987	0.050	0.998	0.056	0.996	0.055
R300/F100		0.990	0.050	0.999	0.046	1.000	0.048		0.986	0.046	0.999	0.056	1.000	0.046
R400/F100		0.998	0.057	1.000	0.052	1.000	0.052		0.986	0.051	0.999	0.054	0.999	0.038
R500/F100		0.997	0.050	0.999	0.046	1.000	0.035		0.984	0.042	1.000	0.047	1.000	0.042

R100/F100	R: Beta (4, 1) F: Beta (4, 1)	0.988	0.048	0.998	0.061	0.998	0.058	R: Beta (4, 0.5) F: Beta (4, 0.5)	0.986	0.046	0.999	0.044	0.997	0.061
R200/F100		0.998	0.052	1.000	0.058	1.000	0.067		0.996	0.056	1.000	0.062	1.000	0.041
R300/F100		0.997	0.055	1.000	0.049	0.999	0.045		0.999	0.057	1.000	0.058	1.000	0.056
R400/F100		0.998	0.048	1.000	0.055	1.000	0.050		0.998	0.057	1.000	0.046	1.000	0.039
R500/F100		0.998	0.051	1.000	0.059	1.000	0.045		0.996	0.047	1.000	0.052	1.000	0.047

R100/F100	R: Beta (1, 4) F: Beta (4, 1)	0.991	0.071	0.996	0.054	0.999	0.069	R: Beta (0.5, 4) F: Beta (4, 0.5)	0.994	0.092	0.997	0.074	0.999	0.08
R200/F100		0.998	0.069	1.000	0.060	1.000	0.071		0.998	0.114	1.000	0.080	1.000	0.062
R300/F100		0.998	0.071	0.999	0.072	1.000	0.059		1.000	0.121	0.999	0.084	1.000	0.073
R400/F100		1.000	0.082	1.000	0.077	1.000	0.076		0.999	0.114	1.000	0.084	1.000	0.056
R500/F100		1.000	0.077	1.000	0.074	1.000	0.055		1.000	0.103	1.000	0.092	1.000	0.083

R100/F100	R: Beta (4, 1) F: Beta (1, 4)	0.958	0.054	0.992	0.073	0.993	0.070	R: Beta (4, 0.5) F: Beta (0.5, 4)	0.939	0.079	0.986	0.081	0.995	0.077
R200/F100		0.981	0.084	0.999	0.076	1.000	0.075		0.966	0.099	0.996	0.086	0.999	0.06
R300/F100		0.978	0.076	0.999	0.063	1.000	0.055		0.972	0.104	1.000	0.106	1.000	0.069
R400/F100		0.991	0.093	0.999	0.061	1.000	0.050		0.982	0.122	0.998	0.081	0.999	0.071
R500/F100		0.990	0.088	0.999	0.073	1.000	0.050		0.970	0.131	0.999	0.095	1.000	0.068

R100/F100	R: N (0, 1) F: N (0, 1)	0.979	0.058	0.997	0.054	1.000	0.063							
R200/F100		0.996	0.051	1.000	0.049	1.000	0.053							
R300/F100		0.998	0.057	0.999	0.060	1.000	0.048							
R400/F100		0.995	0.045	1.000	0.068	1.000	0.044							
R500/F100		0.999	0.061	0.999	0.061	1.000	0.045							

**Table 4:** The mean of Type I error rates and power of MIMIC model for detecting medium uniform-DIF in 10-item scale.

	Distributions for *θ*	3 categories		5 categories		7 categories		Distributions for *θ*	3 categories		5 categories		7 categories	
		Power	Alpha	Power	Alpha	Power	Alpha		Power	Alpha	Power	Alpha	Power	Alpha
R100/F100	R: N (0, 1) F: Beta (1, 4)	0.665	0.051	0.740	0.072	0.780	0.052	R: N (0, 1) F: Beta (0.5, 4)	0.671	0.068	0.768	0.061	0.803	0.062
R200/F100		0.777	0.054	0.884	0.074	0.885	0.055		0.777	0.079	0.883	0.066	0.881	0.068
R300/F100		0.827	0.050	0.894	0.054	0.927	0.053		0.817	0.084	0.894	0.062	0.920	0.051
R400/F100		0.833	0.056	0.911	0.054	0.937	0.053		0.832	0.061	0.913	0.073	0.946	0.066
R500/F100		0.842	0.062	0.929	0.043	0.949	0.061		0.839	0.072	0.930	0.071	0.950	0.050

R100/F100	R: N (0, 1) F: Beta (4, 1)	0.710	0.053	0.776	0.065	0.790	0.049	R: N (0, 1) F: Beta (4, 0.5)	0.743	0.058	0.789	0.061	0.829	0.062
R200/F100		0.823	0.049	0.901	0.066	0.902	0.057		0.840	0.065	0.888	0.069	0.917	0.074
R300/F100		0.857	0.056	0.907	0.062	0.938	0.054		0.868	0.065	0.914	0.058	0.937	0.061
R400/F100		0.877	0.061	0.925	0.054	0.949	0.058		0.867	0.081	0.947	0.068	0.952	0.077
R500/F100		0.901	0.060	0.946	0.049	0.956	0.05		0.906	0.056	0.940	0.059	0.966	0.063

R100/F100	R: Beta (1, 4) F: Beta (1, 4)	0.708	0.058	0.756	0.051	0.807	0.058	R: Beta (0.5, 4) F: Beta (0.5, 4)	0.673	0.059	0.780	0.048	0.812	0.062
R200/F100		0.793	0.042	0.870	0.062	0.880	0.052		0.786	0.053	0.862	0.049	0.881	0.053
R300/F100		0.837	0.047	0.929	0.050	0.929	0.054		0.837	0.049	0.891	0.049	0.913	0.057
R400/F100		0.841	0.050	0.911	0.057	0.925	0.044		0.833	0.045	0.921	0.052	0.938	0.055
R500/F100		0.879	0.068	0.934	0.064	0.958	0.059		0.853	0.043	0.919	0.043	0.940	0.048

R100/F100	R: Beta (4, 1) F: Beta (4, 1)	0.701	0.067	0.783	0.051	0.825	0.057	R: Beta (4, 0.5) F: Beta (4, 0.5)	0.703	0.052	0.788	0.053	0.835	0.048
R200/F100		0.806	0.044	0.882	0.056	0.917	0.056		0.824	0.059	0.896	0.061	0.909	0.052
R300/F100		0.868	0.037	0.934	0.053	0.929	0.048		0.879	0.054	0.926	0.053	0.931	0.053
R400/F100		0.847	0.055	0.931	0.046	0.948	0.042		0.879	0.047	0.940	0.053	0.962	0.043
R500/F100		0.889	0.056	0.935	0.065	0.961	0.064		0.886	0.050	0.948	0.064	0.958	0.046

R100/F100	R: Beta (1, 4) F: Beta (4, 1)	0.701	0.060	0.754	0.064	0.813	0.064	R: Beta (0.5, 4) F: Beta (4, 0.5)	0.687	0.086	0.773	0.077	0.815	0.066
R200/F100		0.809	0.076	0.871	0.071	0.893	0.065		0.813	0.127	0.880	0.083	0.893	0.092
R300/F100		0.857	0.075	0.928	0.057	0.932	0.053		0.842	0.121	0.908	0.081	0.924	0.081
R400/F100		0.833	0.081	0.920	0.066	0.940	0.058		0.845	0.109	0.929	0.080	0.946	0.084
R500/F100		0.896	0.094	0.923	0.080	0.954	0.074		0.872	0.113	0.936	0.092	0.959	0.079

R100/F100	R: Beta (4, 1) F: Beta (1, 4)	0.680	0.088	0.739	0.053	0.798	0.054	R: Beta (4, 0.5) F: Beta (0.5, 4)	0.627	0.095	0.707	0.078	0.779	0.063
R200/F100		0.755	0.068	0.849	0.063	0.865	0.06		0.738	0.098	0.816	0.075	0.860	0.073
R300/F100		0.793	0.056	0.910	0.067	0.915	0.061		0.802	0.133	0.871	0.096	0.891	0.082
R400/F100		0.799	0.079	0.903	0.076	0.913	0.055		0.797	0.135	0.883	0.081	0.906	0.086
R500/F100		0.842	0.093	0.917	0.080	0.939	0.075		0.816	0.108	0.890	0.083	0.917	0.072

R100/F100	R: N (0, 1) F: N (0, 1)	0.696	0.052	0.786	0.054	0.817	0.058							
R200/F100		0.785	0.055	0.855	0.054	0.900	0.05							
R300/F100		0.860	0.054	0.910	0.048	0.926	0.057							
R400/F100		0.869	0.050	0.929	0.062	0.954	0.045							
R500/F100		0.870	0.052	0.922	0.059	0.971	0.055							

**Table 5:** The mean of Type I error rates and power of MIMIC model for detecting severe uniform-DIF in 10-item scale.

	Distributions for *θ*	3 categories		5 categories		7 categories		Distributions for *θ*	3 categories		5 categories		7 categories	
		Power	Alpha	Power	Alpha	Power	Alpha		Power	Alpha	Power	Alpha	Power	Alpha
R100/F100	R: N (0, 1) F: Beta (1, 4)	0.975	0.051	0.998	0.071	1.000	0.051	R: N (0, 1) F: Beta (0.5, 4)	0.969	0.066	0.997	0.061	0.999	0.060
R200/F100		0.991	0.054	1.000	0.075	1.000	0.055		0.986	0.079	0.998	0.065	1.000	0.069
R300/F100		0.992	0.052	0.999	0.054	1.000	0.053		0.992	0.085	0.999	0.062	1.000	0.051
R400/F100		0.997	0.056	1.000	0.054	0.999	0.053		0.990	0.061	1.000	0.073	1.000	0.065
R500/F100		0.993	0.062	1.000	0.043	1.000	0.061		0.990	0.071	1.000	0.071	1.000	0.050

R100/F100	R: N (0, 1) F: Beta (4, 1)	0.989	0.053	0.998	0.064	0.999	0.049	R: N (0, 1) F: Beta (4, 0.5)	0.997	0.057	1.000	0.062	0.999	0.062
R200/F100		0.998	0.050	1.000	0.065	1.000	0.057		1.000	0.065	1.000	0.069	1.000	0.074
R300/F100		1.000	0.056	1.000	0.062	1.000	0.054		1.000	0.065	1.000	0.057	1.000	0.061
R400/F100		0.999	0.062	1.000	0.054	1.000	0.056		1.000	0.080	1.000	0.068	1.000	0.077
R500/F100		1.000	0.061	1.000	0.049	1.000	0.050		1.000	0.056	1.000	0.060	1.000	0.063

R100/F100	R: Beta (1, 4) F: Beta (1, 4)	0.991	0.058	0.997	0.051	0.998	0.059	R: Beta (0.5, 4) F: Beta (0.5, 4)	0.977	0.059	0.995	0.048	0.997	0.061
R200/F100		0.989	0.044	0.999	0.062	1.000	0.051		0.991	0.053	1.000	0.049	0.999	0.053
R300/F100		0.998	0.048	1.000	0.051	0.999	0.054		0.994	0.050	1.000	0.049	0.999	0.057
R400/F100		0.994	0.050	1.000	0.057	1.000	0.045		0.991	0.044	1.000	0.052	1.000	0.055
R500/F100		0.998	0.068	1.000	0.064	1.000	0.059		0.996	0.043	0.999	0.043	1.000	0.048

R100/F100	R: Beta (4, 1) F: Beta (4, 1)	0.992	0.067	0.998	0.049	1.000	0.057	R: Beta (4, 0.5) F: Beta (4, 0.5)	0.998	0.053	1.000	0.054	1.000	0.049
R200/F100		1.000	0.043	1.000	0.056	1.000	0.056		0.999	0.057	1.000	0.061	1.000	0.051
R300/F100		0.998	0.037	1.000	0.053	1.000	0.048		0.998	0.054	1.000	0.053	1.000	0.053
R400/F100		0.997	0.054	1.000	0.046	1.000	0.042		0.997	0.046	1.000	0.053	1.000	0.043
R500/F100		1.000	0.056	1.000	0.065	1.000	0.064		0.998	0.050	0.999	0.063	1.000	0.046

R100/F100	R: Beta (1, 4) F: Beta (4, 1)	0.997	0.059	0.996	0.063	1.000	0.065	R: Beta (0.5, 4) F: Beta (4, 0.5)	0.996	0.086	1.000	0.078	1.000	0.066
R200/F100		1.000	0.078	1.000	0.070	1.000	0.065		0.999	0.127	1.000	0.083	1.000	0.092
R300/F100		1.000	0.076	1.000	0.056	1.000	0.054		1.000	0.121	1.000	0.081	1.000	0.081
R400/F100		0.999	0.081	1.000	0.066	1.000	0.059		1.000	0.110	1.000	0.080	1.000	0.084
R500/F100		1.000	0.094	1.000	0.079	1.000	0.074		1.000	0.114	1.000	0.092	1.000	0.079

R100/F100	R: Beta (4, 1) F: Beta (1, 4)	0.978	0.087	0.994	0.052	0.999	0.056	R: Beta (4, 0.5) F: Beta (0.5, 4)	0.955	0.094	0.989	0.080	0.995	0.063
R200/F100		0.979	0.066	0.997	0.064	1.000	0.060		0.973	0.099	0.997	0.076	0.999	0.072
R300/F100		0.988	0.055	0.999	0.068	1.000	0.061		0.977	0.132	0.997	0.097	0.998	0.082
R400/F100		0.984	0.080	0.998	0.075	1.000	0.055		0.972	0.135	0.999	0.080	1.000	0.085
R500/F100		0.990	0.093	0.998	0.079	1.000	0.075		0.984	0.108	0.999	0.083	1.000	0.072

R100/F100	R: N (0, 1) F: N (0, 1)	0.993	0.052	0.998	0.056	1.000	0.059							
R200/F100		0.996	0.056	0.999	0.054	1.000	0.050							
R300/F100		0.998	0.055	1.000	0.048	1.000	0.057							
R400/F100		0.997	0.049	1.000	0.061	1.000	0.045							
R500/F100		0.996	0.052	1.000	0.059	1.000	0.056							

**Table 6:** Detection of uniform-DIF for GHQ-12 with MIMIC model.

GHQ-12 items		M100/F100	M100/F200	M100/F300	M100/F400	M100/F500
Item 1	Able to concentrate	−	−	−	+	+
Item 2	Lost much sleep	−	−	−	−	−
Item 3	Playing a useful part	−	−	−	−	−
Item 4	Capable of making decisions	−	−	−	−	−
Item 5	Under stress	−	−	−	−	−
Item 6	Could not overcome difficulties	−	−	−	−	−
Item 7	Enjoy your day-to-day activities	−	−	−	−	−
Item 8	Face up to problems	−	−	−	−	−
Item 9	Feeling unhappy and depressed	−	−	−	−	−
Item 10	Losing confidence	−	−	−	−	−
Item 11	Thinking of self as worthless	−	−	−	−	−
Item 12	Feeling reasonably happy	+	+	+	+	+

The plus sign indicates item with DIF and the minus sign indicates item with free-DIF; the letter “M” means male and the letter “F” means female.
